# Identifiability of Bayesian models of perception

**DOI:** 10.1073/pnas.2601013123

**Published:** 2026-09-11

**Authors:** Michael Hahn, Entang Wang, Xue-Xin Wei

**Affiliations:** ^a^ Department of Language Science and Technology, Saarland Informatics Campus, Saarland University, Saarbrücken 66123, Germany; ^b^ Department of Neuroscience, The University of Texas at Austin, Austin, TX 78712; ^c^ Department of Psychology, The University of Texas at Austin, Austin, TX 78712; ^d^ Center for Perceptual Systems, The University of Texas at Austin, Austin, TX 78712; ^e^ Center for Learning and Memory, The University of Texas at Austin, Austin, TX 78712; ^f^ Center for Theoretical and Computational Neuroscience, The University of Texas at Austin, Austin, TX 78712

**Keywords:** Bayesian model, model identifiability, behavior, experimental design, cognition

## Abstract

Inferring the underlying computational processes from behavioral measurements is a fundamental approach in cognitive science and neuroscience. Although Bayesian decision theory has become a major normative framework for modeling perception and cognition, it is unclear to what extent its modeling components (i.e., prior belief, likelihood function, and loss function) can be recovered from behavioral data. Here, we systematically investigated the problem of inferring such Bayesian models from behavioral tasks. We determined the situations under which some components of Bayesian models are systematically confounded, as well as the practical choices in experimental design that can resolve such ambiguity. Overall, our analytical results guarantee in-principle identifiability under broadly applicable conditions, without any a priori knowledge of prior or encoding. Simulations and applications on the basis of behavioral datasets validate that the predictions of this theory apply in realistic settings. Importantly, our results demonstrate that reliable recovery of the model often requires having data from multiple noise levels. This is a crucial insight that will guide future experimental design.

## Significance

Bayesian models represent an important class of models in cognitive science and neuroscience. The model components (prior, likelihood function, loss function) play important roles in understanding perceptual and cognitive processing. So far, it remains unclear whether and how well these components can be inferred from behavioral experiments. Here, we determine the conditions under which the model components can be identified from the data. Our results offer important guidance for the design and interpretation of behavioral experiments for investigating cognition.

In cognitive science and neuroscience, Bayesian decision theory has emerged as a major principled framework for understanding human perception (1–11) and cognition (12–14). This framework provides a normative recipe for modeling behavior by combining three ingredients (15). The first is the prior belief, which is a probability distribution that describes which stimuli the observer a priori considers more or less likely to have appeared, prior to any sensory input. The second is the likelihood function, which describes how likely the sensory observation is to be generated based on a given stimulus. The prior belief and the likelihood function are combined via Bayes’ rule to obtain a posterior probability distribution, i.e., an estimate of how likely each possible stimulus value is to have produced the noisy encoding. The third ingredient is the loss function, an assignment of costs to estimation errors. It may, for instance, be relatively tolerant of small estimation errors, or could penalize all estimation errors equally. Bayesian decision theory prescribes choosing the stimulus that minimizes the posterior expectation of the loss function.

There has been substantial interest in reverse-engineering these components from measured human behavior (5, 6, 16–19). This approach has been applied to the domains of perception (5, 6, 16, 19, 20), sensory-motor processing (17, 21) and, more recently, in neuroeconomics (22, 23). The basic idea is to make inferences about the prior, encoding, and loss functions from behavioral observations. While Bayesian models have been widely used in behavioral sciences, surprisingly, the question of whether Bayesian models are identifiable has not been systematically investigated. It is sometimes believed that the Bayesian approach is underconstrained and inherently degenerate (19), in that multiple different models can give rise to the same behavior. Prior studies have often presupposed some part of the model, as a way of constraining the model fit. As noted in ref. 24, work often fixes one of the three components when estimating observer models, e.g., constraining the encoding by measuring discriminability (e.g., ref. 25), measuring naturalistic priors (e.g., ref. 26), imposing a loss function experimentally (e.g., ref. 27), or assuming both the encoding and loss function were given (28). So far, it remains unknown whether the model components of Bayesian models are actually identifiable from behavioral data. Perhaps not surprisingly, the lack of a theoretical foundation in model recovery has led to serious criticism of the Bayesian approach (29–31).

Understanding whether a computational approach can recover the ground truth from empirical data is a basic problem in virtually all areas of science that use model-based inference, not limited to Bayesian models. In statistical estimation, this problem can be formulated as the consistency of the estimator (32, 33), and is fairly well understood for simple estimation problems, e.g., estimating the mean of a Gaussian random variable from samples. However, for more complex models, it can be challenging to precisely understand the conditions under which the models are identifiable (34). The question of model identifiability has drawn more attention in economics (35, 36) and systems biology (37, 38). However, these results are not directly applicable for Bayesian models of cognition. The lack of understanding of the identifiability of Bayesian models poses a fundamental challenge for interpreting empirical observations from experiments.

We fill this critical gap by developing a rigorous theory of the identifiability of Bayesian models of perceptual decision-making from behavioral data, focusing on estimation tasks. On the one hand, we find that, indeed, Bayesian models have substantial degeneracy, in that simple experimental setups will, in systematic ways, fail to fully identify the three model components. On the other hand, we rigorously prove that, using more carefully designed experimental setups, e.g., by experimentally varying the magnitude of neural noise between trials, one can systematically eliminate this degeneracy. Remarkably, we find that Bayesian models are identifiable without any of the parametric assumptions often made for the encoding or the prior.

A crucial insight from these results is that it is beneficial to collect measurements at multiple levels of sensory/neural noise in experiments in order to recover the model. Importantly, collecting measurements at multiple levels of sensory or neural noise is already done in some experimental work and can be achieved, e.g., by varying exposure duration (e.g., refs. 22, 39, and 40), contrast (e.g., ref. 41), or memory delay (e.g., ref. 42). We demonstrate that this practical choice is in fact essential to identifiability, and should be broadly adopted whenever identifying prior and loss function are a concern. Simulations and applications on the basis of a series of previously published behavioral datasets validate the theoretical results in realistic settings. Our results have important implications in interpreting results from fitting Bayesian observer models to empirical data and provide guiding principles for designing experiments to understand how perceptual decisions are computed in the brain based on behavioral measurements. A preliminary version of this work was presented earlier (43).

## 1. Results

**Fig. 1.**
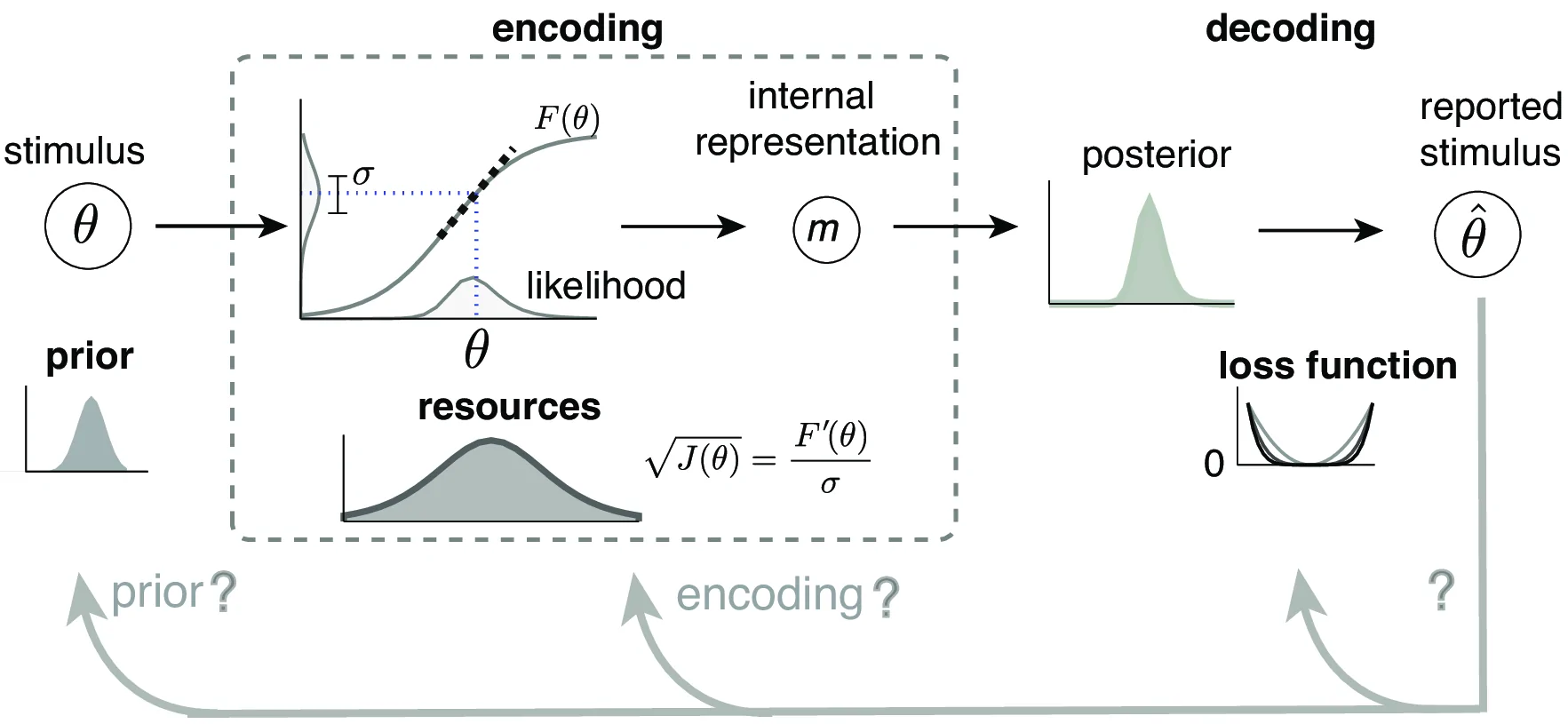
The Bayesian model framework and the problem of model identification. For a given stimulus variable θ, the neural code is modeled as a one-dimensional encoding function corrupted by Gaussian sensory noise. The sensitivity of the population activity to the stimulus is described by the Fisher information. Given an encoding m obtained from the encoding function F and sensory (neural) noise, Bayesian inference is used to decode a point estimate $\hat{\theta }$. Specifically, for decoding, the observer computes the posterior distribution over the stimulus variable by combining the likelihood function and the prior distribution, and then seeks to minimize a loss function to decode the stimulus. Here, we focus on a family of $L_{p}$ loss functions that covers typical loss functions considered in the literature. This decoded stimulus $\hat{\theta }$ is assumed to reflect the stimulus reported by the observer in the continuous estimation task, up to corruption by motor noise. The identifiability problem asks to what extent it is possible to recover the three components of the Bayesian model—prior, encoding resources (Fisher information), and loss function—from the behavioral responses.

We will primarily study models of continuous estimation tasks. For example, consider a task where, on each trial, the subject is presented with an oriented bar, and needs to report the perceived orientation. Following previous studies (8, 11, 22, 44–46), we model the neural processing of a stimulus variable θ (for example, the orientation of a bar) as a cascade of encoding and decoding computations. In particular, we model the encoding of a one-dimensional variable using a general nonlinear transformation F, subject to additive noise (Fig. 1):

[1] $$\begin{array}{c} m=F(\theta )+\delta , \end{array}$$

where δ represents the sensory noise, which is assumed to have a Gaussian distribution with variance $\sigma^{2}$ describing the magnitude of sensory noise (internal noise). Sensory noise arises due to limitations in the number of spikes or cognitive resources. While simple, this model provides a good approximation of more complex neural population coding models with realistic noise assumptions such as Poisson spiking noise (47). We focus on the 1-D case since most existing Bayesian models deal with a 1-D stimulus variable, but we expect the theory to extend to the multivariate case. The Bayesian framework contains several important components. The first component is the encoding function F. The encoding function determines how coding resources are allocated, i.e., the slope of the encoding function F for a given stimulus (normalized by the SD of the noise) determines the Fisher information: $\sqrt{J(\theta )}=\frac{F^{\prime }\left(\theta \right)}{\sigma }$. Fisher information quantifies the coding resources allocated to the given stimulus and has direct implications in terms of discriminability and just-noticeable difference (JND) (48). Uniform Fisher information implies an equal JND space. For a stimulus variable with nonequal JND, the slope of encoding function F captures the nonuniform allocation of coding resources (8, 11). A larger slope of the encoding function (i.e., more coding resources) should lead to better discriminability and a smaller JND. Thus, later we will also refer to $\sqrt{J(\theta )}$ as the “resource allocation or resources” The second component is the prior over the stimulus variable, given by a probability distribution $p_{\mathrm{prior}}\left(\theta \right)$. The prior distribution summarizes the knowledge that the observer has about the stimulus variables before the sensory measurement was obtained. The third component is the loss function, which quantifies the cost associated with the inaccuracy in estimating the stimulus. Popular loss functions involve 0–1 loss, squared error loss, absolute distance, which are members of the general family of $L_{p}$ loss functions. We will focus on loss functions from this family. Under the Bayesian framework, the observer obtains a noisy sensory measurement on each trial and computes a posterior distribution by multiplying the prior and likelihood. The reported stimulus by the observer is modeled as the stimulus that minimizes the loss function.

We are interested in understanding whether the components of the Bayesian model can be recovered from behavioral responses. While our analytical results on the Bayesian models below focus on the small-noise regime, extensive numerical simulations are used to verify that conclusions hold more generally.

### 1.1. Prior and Encoding Can be Identified when the Loss Function Is a Known $L_{p}$ Loss.

Suppose that we have collected behavioral responses from a sufficiently large number of trials in an estimation task from a Bayesian observer. On each trial, a particular stimulus θ was presented, and the observer reported the stimulus that they perceived. We first consider the case that the loss function is known to be the squared error loss (a popular loss function assumed in the literature). We ask whether one can recover the prior and encoding from the behavior responses. Theoretically, we find that the answer is always “yes,” under mild assumptions on the smoothness of the resource allocation and the prior, and small sensory noise.

First, resource allocation (i.e., encoding) can be recovered by comparing the overlap between response distributions elicited by a given stimulus θ and a nearby stimulus θ+Δθ. Intuitively, a smaller overlap indicates that more coding resource is allocated around θ. We show that, mathematically, this idea can be used to exactly recover the resource allocation (*Theorem* 3.1).

Second, the prior distribution can be identified by further considering the relationship between the estimation bias and the slope of the prior, which largely follows a one-to-one correspondence under a squared error loss:

[2] $$\begin{array}{c} \text{Bias}\left(\theta \right)=\frac{1}{J(\theta )}{\left(\log p_{\mathrm{prior}}\left(\theta \right)\right)}^{\prime }+{\left(\frac{1}{J(\theta )}\right)}^{\prime }, \end{array}$$

when $J\left(\theta \right)=\frac{F^{\prime }{\left(\theta \right)}^{2}}{\sigma^{2}}$ denotes the Fisher information, which represents the amount of resources for encoding θ. Once the resource allocation has been identified, the prior can be identified from the bias via Eq. **2**.

Notably, the same conclusion holds when the known loss function belongs to the general $L_{p}$ family, i.e., the observer’s loss is determined by a power function of the absolute error. This includes loss functions that are commonly adopted in the literature, e.g., MAP (p→0), median (p=1), or mean squared error (p=2).

These analytical results are derived in the regime where sensory noise is small. To test their validity in realistic settings, we used a general fitting procedure that represents encoding and prior on a discretized grid, and optimizes these and the magnitude of noise to maximize trial-by-trial likelihood of observed data. Results based on extensive numerical simulations support our analytical results. We find that the model components are closely recovered when noise is small. Together, these results provide theoretical justifications of empirically reverse-engineering the prior and encoding when we have good knowledge of the observer’s decision rule.

**Fig. 2.**
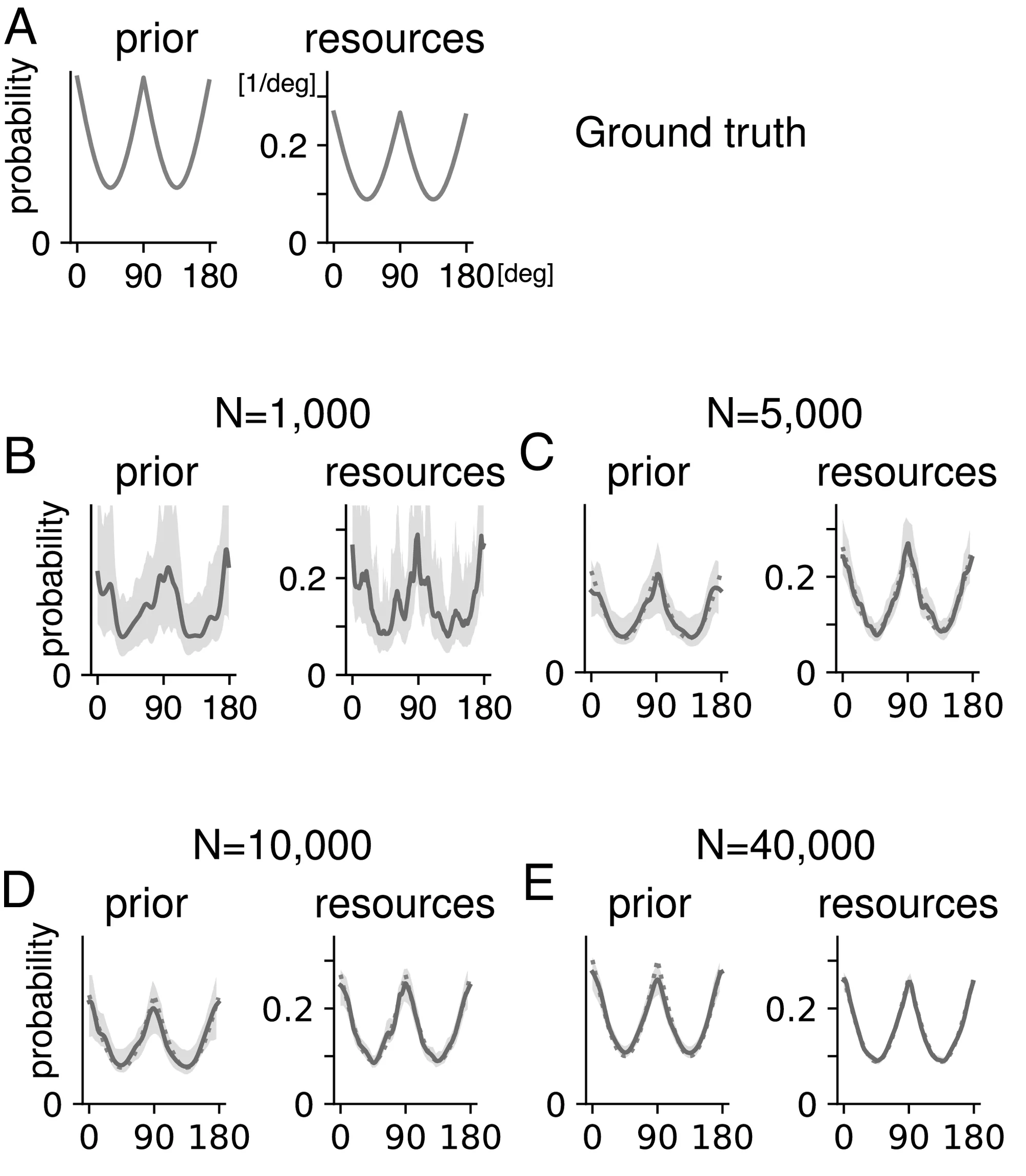
Prior and encoding can be identified when the loss function is known. Here we illustrate the identifiability of prior and encoding resources when the loss function is known using an example. In particular, we show that nonparametric priors and encodings with $L_{2}$ loss can be identified when sufficiently many trials at small sensory noise are available. (*A*) The ground truth model. We show the prior as a probability density, and the encoding resources $\sqrt{J(\theta )}$ (with units [1/deg]). Here, both are higher for the cardinal orientations (0 and 90 degree), and lower for the oblique orientations (45 and 135 degree). The loss function is the squared error loss, and is assumed to be known. (*B*) We simulated a dataset with 1,000 trials by randomly drawing 1,000 samples from the generative model in (*A*). We then fit the Bayesian model to this simulated dataset assuming a squared error loss function. We show point estimates (solid), the ground truth (dotted), and bootstrapped 95% confidence bands. The results show that inferred prior and resources capture the basic patterns of the ground truth. (*C*–*E*) Similar to (*B*), but for even larger sample sizes. Increasing the sample size leads to more accurate model recovery. *SI Appendix*, Figs. S35 and S36, has results for further models and other noise magnitudes.

Fig. 2 illustrates one simulation example, inspired by data from orientation estimation tasks (11, 39, 49). In this case, the ground truth prior is higher at the cardinal orientations and the encoding is such that cardinal orientations are encoded better, consistent with the efficient coding hypothesis (44, 50). We found that, with 1,000 trials, the basic shape of the prior and encoding can be readily recovered. As expected, the recovery improves when using more trials. Notably, this suggests that one can leverage estimation tasks and the Bayesian framework to effectively infer the allocation of encoding resources. This observation may have important implications for experimental work. The coding resource is the inverse of the discrimination threshold, which is an important behavioral measure in psychophysics that is often estimated using binary classification tasks. One limitation of inferring discriminability using binary classification tasks is that it often requires a large number of trials. Our simulation results suggest that the continuous estimation paradigm offers a viable alternative in inferring stimulus-dependent coding resources for one-dimensional stimulus variables. Later we will test this procedure further using experimental data.

When noise increases, the allocation of the coding resource (i.e., encoding) can still be recovered, though the prior distribution may become difficult to recover as large noise may wash out details of the prior (*SI Appendix*, Fig. S13). These results have a further implication for future experiments, that is, the inclusion of conditions at small sensory noise could be important for identifying the prior. For certain vision experiments, sensory noise may be reduced by increasing the contrast or the presentation time of the visual stimuli.

### 1.2. Prior and Loss Function Can be Systematically Confounded when the Loss Function Is Unknown.

While, in some experiments, we may have a priori good knowledge about the loss function used by the observer, this does not hold generally. Thus, we next consider the question of model identifiability under a more general setting for which the loss function is unknown. For now, we will assume that the magnitude σ of sensory noise is small and shared across all trials, and that the loss function belongs to the general $L_{p}$ loss family. Importantly, neither the magnitude of sensory noise nor the exponent p are known a priori.

Using the same theoretical arguments as above, the encoding (i.e., Fisher information) can be well identified even when the loss is unknown. However, for the loss function and prior, we find that they can be systematically confounded. That is, models with different combinations of prior and loss function may explain the data equally well. In particular, when the sensory noise is small, a model (model 1) with prior $p_{\mathrm{prior}}^{\left(1\right)}$ and loss function exponent $p_{1}$ is indistinguishable from any model (model 2) with the same encoding but a different loss function exponent $p_{2}$, and with a prior $p_{\mathrm{prior}}^{\left(2\right)}\left(\theta \right)$ given as

[3] $$\begin{array}{c} p_{\mathrm{prior}}^{\left(2\right)}\left(\theta \right)\propto p_{\mathrm{prior}}^{\left(1\right)}\left(\theta \right)\cdot J{\left(\theta \right)}^{\frac{p_{2}-p_{1}}{4}}, \end{array}$$

(see *SI Appendix*, *Theorem* S9 for formal proof). The MAP estimator (p→0 in the loss function) is special; here, the exponent needs to be replaced by −1 rather than 0 in this formula. Thus, prior and loss function can be systematically confounded in experiments with only one level of small sensory noise.

**Fig. 3.**
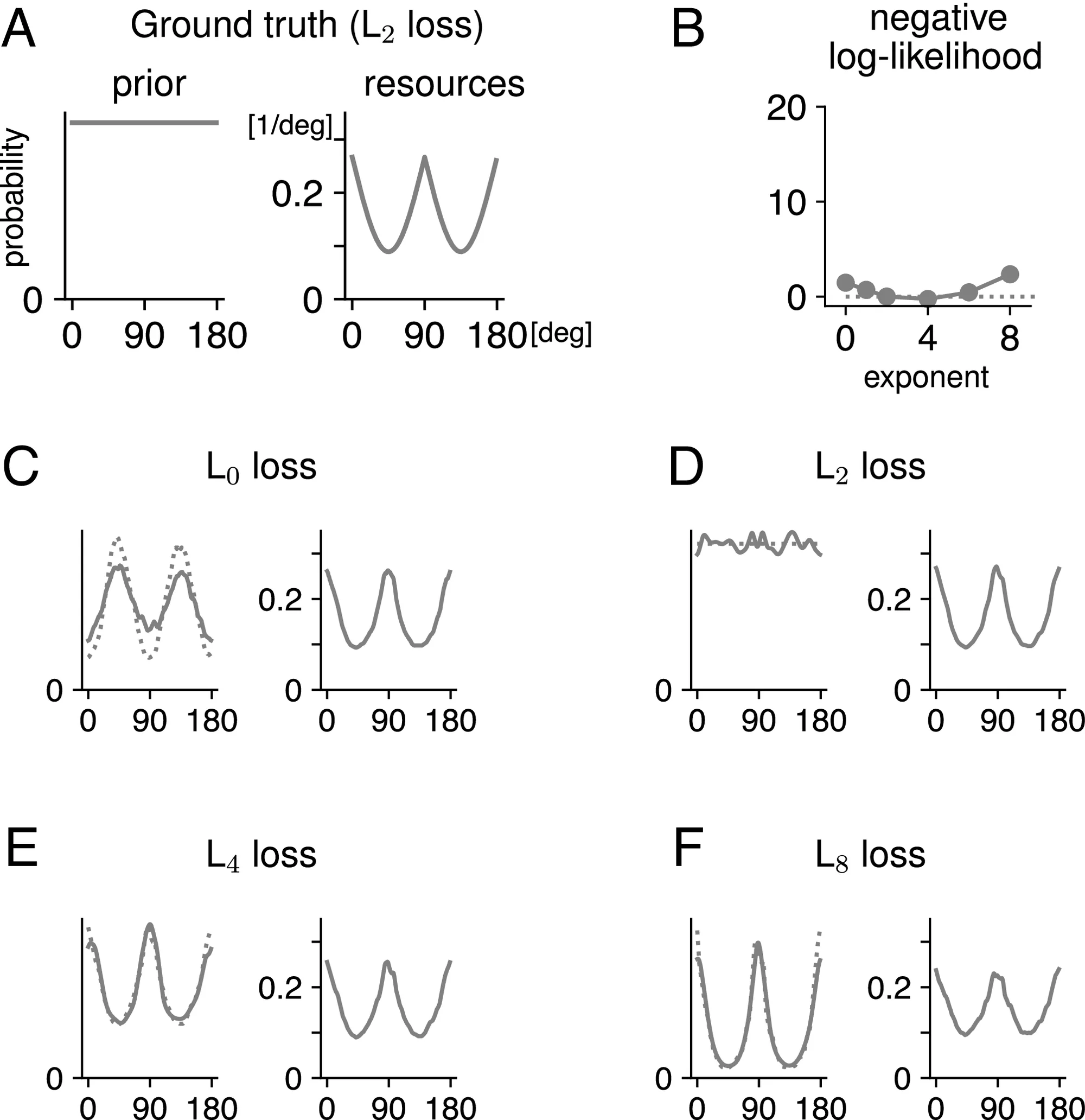
Prior and loss function can be confounded when both are unknown. We use a simulation example to demonstrate that simple experimental designs are typically sufficient for identifying the encoding, but cannot recover prior and loss function. (*A*) The ground truth model component, with a uniform prior and nonuniform encoding resources. (*B*) We generate a dataset with 10K trials data assuming the model in (*A*). We then fit the data to models assuming different loss functions parameterized by a $L_{p}$ family of functions. Model fit (heldout negative log likelihood, NLL) does not single out the ground-truth exponent, making it difficult to adjudicate between different models. (*C*–*F*) The best fitted prior and encoding resources when assuming different loss functions. In each case, we plot both an idealized curve predicted by the theory (dotted, Eq. **3**), and a fit generated by the fitting procedure (solid). We observe that, while the encoding is recovered throughout, the prior is recovered incorrectly (in accordance with Eq. **3**) when the loss function is misspecified. Overall, this means that the prior and loss function are confounded.

These points are illustrated using an example in Fig. 3. We generate synthetic datasets based on the same ground truth model as in Fig. 2: higher prior density and encoding precision for cardinal orientations, and squared error loss functions. We then fit the Bayesian model assuming the correct loss function. Fig. 3*D* shows that the fit can well recover the prior and the encoding model, as expected. However, because the loss function is not known in this problem, one may also fit a model assuming a different loss function, e.g., $L_{0}$ (MAP estimator) or $L_{8}$ loss. Fitting to the data with those functions, one finds that the encoding is again well recovered. However, the fitted prior in this case has substantial lower (p=0) or higher (p=4,8) density at the cardinal orientations (Fig. 3*C*, *E*, and *F*). Which prior reflects the ground truth then? One may attempt to use the log-likelihood of the model fits to adjudicate between the models. However, models with different loss functions explain the data equally well (Fig. 3*B*). Thus, in this case, it is impossible to identify the model.

While Eq. **3** shows that the prior and loss functions generally cannot be identified when the loss is unknown, there is a special case where the prior can be identified: when the Fisher information is uniform (i.e., the stimulus space is an equal JND space). In this case, according to Eq. **3**, the fitted prior is identical under different $L_{p}$ loss functions, thus can be identified, even though the loss function may remain unidentifiable.

### 1.3. Leveraging Behavioral Responses Based on Multiple Sensory Noise Levels Makes Most Models Identifiable.

At first sight, the results described above may suggest a somewhat pessimistic view of the ability to identify Bayesian observer models when neither the prior nor the loss function is known a priori. However, it is important to realize that the setting studied above assumes that one only collects the behavioral responses of the observer under a single sensory noise condition, i.e., the magnitude of the sensory noise is shared in all trials. Crucially, we find that this degeneracy in identifiability can typically be avoided by experimental setups where the amount of sensory noise is not shared across all trials. As mentioned above, sensory noise may be manipulated to a certain extent by varying the length of the stimulus presentation time or the contrast of the stimulus.

Importantly, our theoretical analysis shows that the components of Bayesian observer models are usually identifiable if response distributions based on multiple small sensory noise levels and a sufficiently large number of trials are available, across the family of $L_{p}$ loss functions (*Theorem* 3.3 in *Materials and Methods*). Importantly, the experimenter does not need to know a priori how the experimental manipulation (e.g., changing stimulus presentation) maps onto sensory noise magnitudes. It is only required that the manipulation does lead to a change in the noise magnitudes. Different combinations of prior and loss function often make different predictions regarding how the biases should change with the sensory noise levels, which enables ruling out non-ground-truth loss function exponent (*SI Appendix*, Fig. S12).

**Fig. 4.**
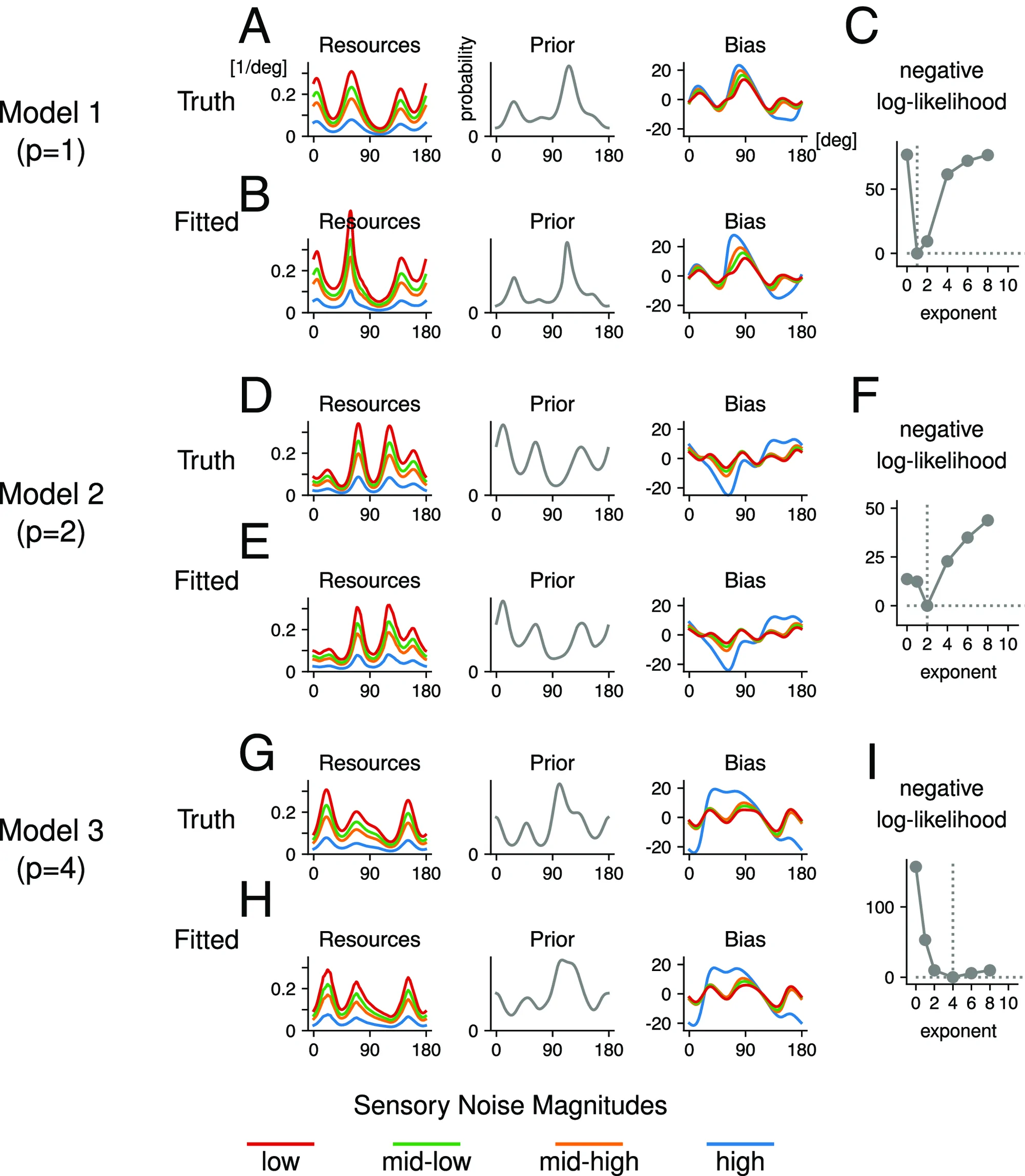
Simulation examples showing that random models can be identified with data from multiple noise levels. Here we show model recovery results from three randomly sampled models, on simulated datasets with 10K trials each. Responses are sampled at four levels of sensory noise: low (red), mid-low (green), mid-high (orange), and high (blue). The sensory noise magnitudes are derived from the dataset collected by de Gardelle et al. (39), but resource allocations and prior are randomly generated. (*A*) The encoding resources, prior distribution of the ground truth model used in the simulation, as well as the predicted behavioral biases of the Bayesian observer. The ground truth loss function is $L_{1}$ for this model. (*B*) The inferred resources, inferred prior, and biases of the best-fit model. (*C*) The negative log-likelihood (NLL) corresponding to the best-fit model under $L_{p}$ loss functions with different exponents. (*D*–*F*) Similar to (*A*–*C*), but for a model with $L_{2}$ loss function. (*G*–*I*) Similar to (*A*–*C*), but for a model with $L_{4}$ loss function.

To validate these theoretical results numerically, we generate many random models with smooth encoding functions and smooth prior density functions, together with the exponent p of the $L_{p}$ loss function. For each model, we generate synthetic datasets by sampling behavioral responses based on multiple noise levels, and then investigate whether the model components can be recovered from the simulated data. In these numerical simulations with experimentally relevant sample sizes, we consistently find that the model components (i.e., prior, encoding, and exponent of the loss function) can be reliably identified when behavioral responses from multiple noise levels are available. Fig. 4 shows three examples of randomly generated models. See *SI Appendix*, section S4.4 for further examples across loss functions.

Psychophysical experiments are often limited by the number of trials. In light of our results above, an important question is, when the total number of trials is constrained, whether collecting behavioral responses at multiple noise levels (e.g., using visual stimuli with different contrast levels) would benefit the identifiability of models. To address this question, we systematically compared the ability to recover the model with a fixed total number of trials, while allowing the number of noise levels to vary in simulations. We perform this analysis across four different combinations of uniform or periodic priors and encodings, across combinations of 1, 2, or 4 noise levels, and different numbers of trials, from 2,000 to 40,000.

**Fig. 5.**
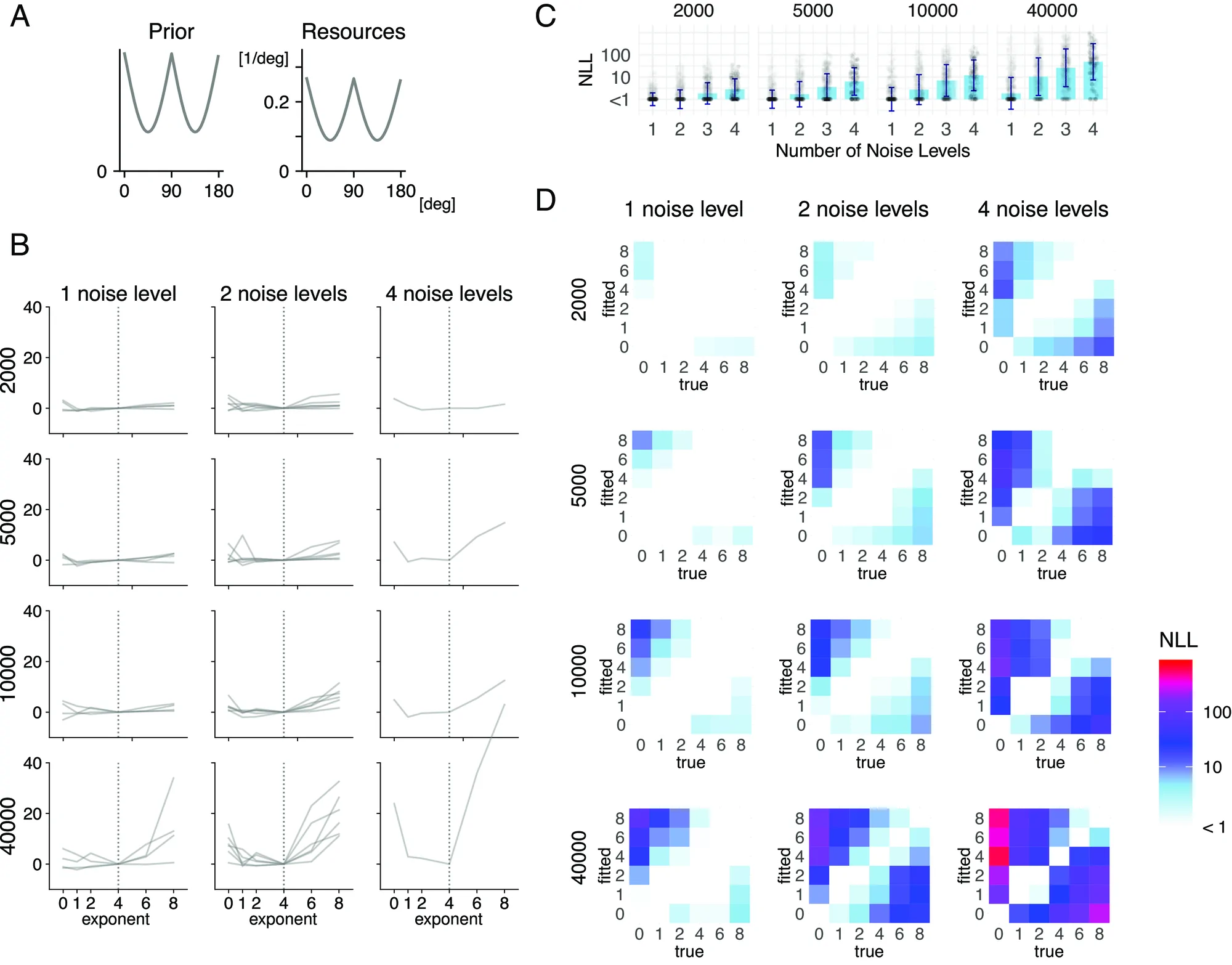
Example simulations showing the benefit of having behavioral responses from multiple noise levels for identifiability. (*A*) We simulated data with periodic prior and encoding at p=4. (*B*) Distinguishing loss functions, with an increasing number of sensory noise levels (columns: 1, 2, 4 levels) and an increasing number of trials (rows: 2K, 5K, 10K, 40K trials). Each line represents one synthetic dataset. The y-axis indicates model fit as quantified by heldout NLL relative to the ground-truth loss. With data from a single level of noise, even a dataset with 40K trials is not always sufficient for identifying the loss. In contrast, and in accordance with the theory, low and high exponents can be reliably distinguished using data from sufficiently many levels of sensory noise. (*C*) Summary across loss exponents. We repeated this experiment for a total of four synthetic combinations of prior and encoding (*Materials and Methods*), for all exponents p=0,1,2,4,6,8. Each dot indicates a combination of model, ground-truth exponent p, and non-ground-truth exponent $p^{\prime }\neq p$ used for fitting. Bars denote medians; error bars indicate SD (on logarithmic scale). Despite substantial variability, increasing the number of noise levels tends to increase identifiability. Sample size: depending on the number of noise levels, each bar has 480 (1 noise level), 720 (2 levels), 480 (3 levels), 120 (4 levels) data points. (*D*) For each pair of ground-truth exponent p and exponent used in fitting $p^{\prime }$, we computed the increase in NLL when fitting with $p^{\prime }$ as compared to p, for four different synthetic combinations of prior and encoding (*Materials and Methods*), with varying numbers of trials and noise levels. Heatmaps plot the median across model runs. Depending on the number of noise levels, each cell reflects 16 (1 noise level), 24 (2 levels), 4 (4 levels) data points. High NLL increase off the diagonal indicates better identifiability of the loss function. Increasing the number of noise levels is highly effective in increasing identifiability.

These results are summarized in Fig. 5. We find that, for a given number of trials, increasing the number of noise levels is generally effective in removing the degeneracy found above. At one level of noise, the identifiability of the loss functions is variable: in some cases, particularly when p is small, a single noise level at intermediate noise already provides sufficient signal for constraining or even identifying p. This suggests that the response distribution obtained from finite noise may contain extra information of the loss functions compared to that of small noise. However, based on the simulations, different exponents of the loss function typically yield similar data likelihoods under experimentally realistic sample sizes. The number of trials needed to faithfully identify the model with one noise level may not be feasible in experiments. When including 2 levels of noise, identifiability is overall increased, in particular when high and low levels of noise are combined (*SI Appendix*, Fig. S14). Furthermore, we found that increasing the number of noise levels further beyond 2 was highly effective in further improving identifiability. With 4 levels of sensory noise, the NLL is steep and generally singles out the ground-truth p, or exponents close to it. Fig. 5*D* suggests that increasing the number of noise levels from one to four may be better for identifiability than increasing the number of trials by a factor of two or more. See *SI Appendix*, sections S4.2 and S4.3 for more simulation results. An important consequence of these results for experimental design is that increasing the number of noise levels can be substantially more efficient for identifiability than increasing the number of trials.

### 1.4. Confronting Models that Are Difficult to Identify Via Adaptation to Different Stimulus Statistics.

We caution that there still exist models for which even having behavioral responses corresponding to multiple noise levels does not ensure identifiability. However, in a mathematical sense, such models are rare and exceptional. We theoretically analyze the probability of encountering such models in the space of all models. Under natural Gaussian-process-based notions of measure or volume on the space of models, the degenerate models have measure 0 (*Theorem* 3.3, *Materials and Methods*). Note that this theoretical result is consistent with our numerical simulations above, which show that most randomly sampled models can be well identified. Mathematically, the exceptional set consists of models where the encoding and the prior jointly satisfy a particular differential equation.

While the identifiability guarantee is valid for all models except a set of measure zero, this exceptional set may nonetheless include models of real-world relevance. In particular, for commonly considered models for scalar variables where the encoding is based on Weber’s law (6, 51, 52) (i.e., the discrimination threshold increases in proportion to the stimulus magnitude), and the prior is lognormal (i.e., normal in the sensory space), an analytical calculation shows that this model belongs to the exceptional set. Interestingly, for this model, the loss function and prior are entangled regardless of the number of noise levels based on which the behavioral responses are collected (*SI Appendix*, section S1.1).

These results pose a challenge to experimental work, i.e., how might one still recover the loss function and the prior in this situation? We find that this ambiguity may be resolved by adapting the system to different contexts with different stimulus statistics to induce a change of the encoding or the prior. As the exceptional set has measure zero, almost all short-term stimulus priors would cause the overall model to exit the exceptional set. This holds when the prior and encoding adapt to the short-term stimulus statistics simultaneously, or when only one of the two adapts (see *SI Appendix*, section S2.7 for a detailed discussion).

### 1.5. Commonly Considered Motor Noise in Estimation Tasks Does Not Affect the Identifiability.

Estimation tasks typically involve the subject reproducing the stimulus variable, e.g., by adjusting a knob to report the perceived orientation of the stimulus. In such reproduction paradigms, motor noise, although generally small in many tasks, may contribute to the behavior response and may need to be explicitly modeled. Empirically, motor noise in these tasks is often modeled as Gaussian additive noise. We seek to understand whether motor noise will hinder the ability to identify the model. We find that adding Gaussian additive motor noise, more generally, symmetric additive noise, does not impact our result (*Theorem* 3.4 in *Materials and Methods*): sensory and motor sources of noise can be dissociated when a sufficient number of trials are available.

### 1.6. Applications to Experimental Datasets.

Next, we use experimental data to test three predictions from our theory. First, the encoding can be easily identified from data from continuous estimation tasks, even with data from only one noise level. Second, the prior distribution and loss function are confounded when data from only one noise level is available; however, for most models, they can be deconfounded by collecting data from multiple noise levels. Third, there exist special models that are difficult to identify even with data from multiple noise levels. We test these predictions on two published datasets. The dataset collected by de Gardelle et al. (39) includes multiple noise levels. The dataset collected by Remington et al. (51) exemplifies the exceptional setup of an encoding based on Weber’s law. As we show below, the results from analyzing the experimental data provide strong support for our theory. These results also suggest experiments for future work.

#### 1.6.1. Application 1: Inferring the stimulus-dependent neural resources from orientation estimation data.

We first test whether the encoding can be estimated reliably using behavioral measurements from continuous estimation tasks where independent evidence from prior research is available (16, 54). To do so, we study a dataset collected by de Gardelle et al. (39), which measured behavioral responses of human observers in an orientation-reproduction paradigm. In this experiment, on each trial, the observer was presented an oriented Gabor stimulus and then was asked to reproduce the perceived orientation. The presentation time of the Gabor stimulus was systematically manipulated (20, 40, 80, 160, and 1,000 ms; five conditions in total). We hypothesize that reducing the presentation time of the stimulus would increase the magnitude of the sensory noise, and reduce the Fisher information at each stimulus orientation proportionally.

**Fig. 6.**
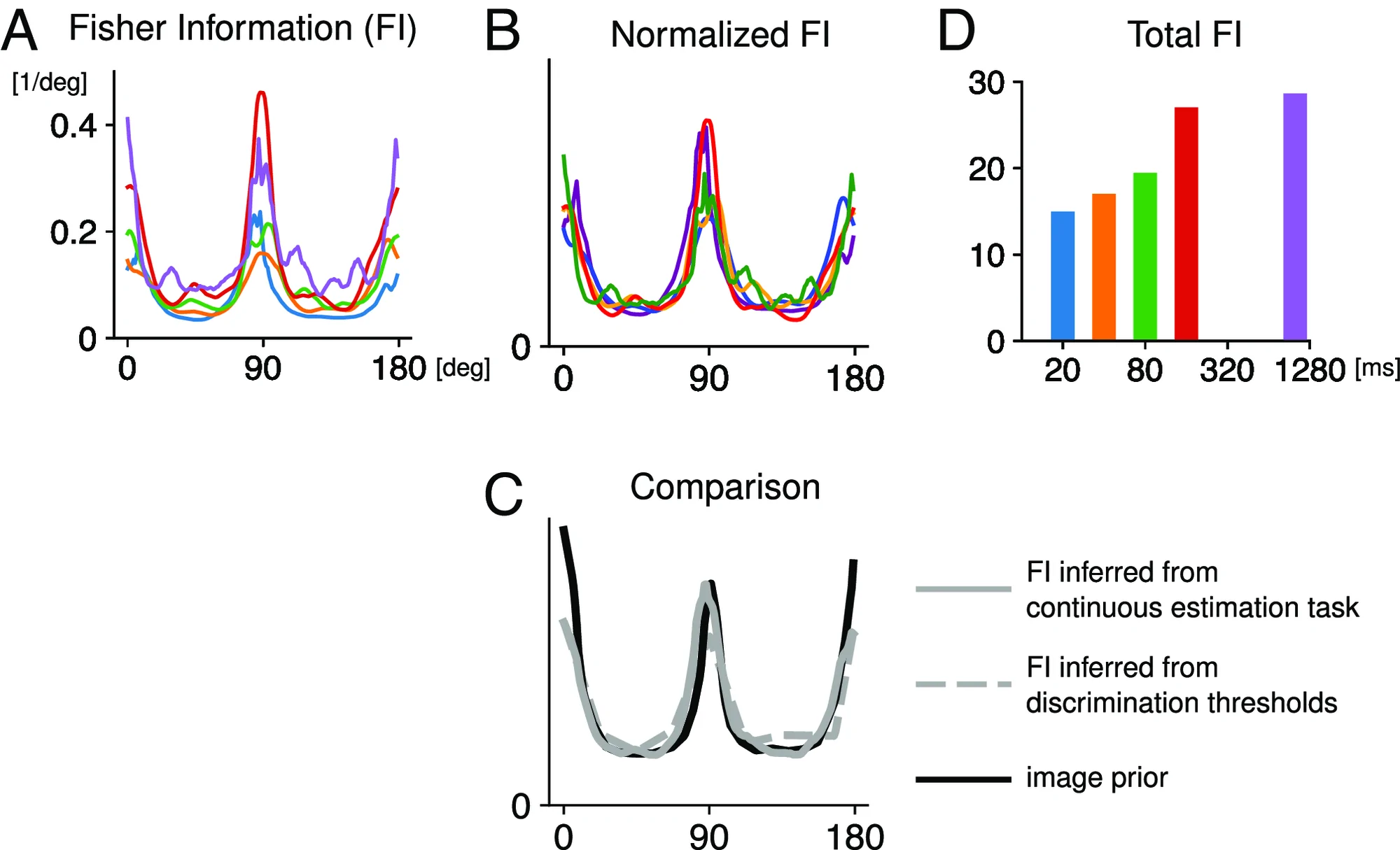
Application to experimental data: inferring the coding resource allocation for visual orientation from behavioral data. We seek to identify the encoding for orientation of Gabor arrays from human estimation data [collected by de Gardelle et al. (39)]. We report the results of fitting the encoding individually on 1K trials from each of the five exposure durations. (*A*) The square root of the Fisher information, $\sqrt{J(\theta )}$ for each of the five experimental conditions. Each condition (indicated with a distinct color) corresponds to a different amount of stimulus presentation time, i.e., 20 (blue), 40 (orange), 80 (green), 160 (red), and 1,000 ms (purple). (*B*) The normalized Fisher information, corresponding to the slope of the transfer function $F^{\prime }\left(\theta \right)$, shows high consistency across the five conditions. For each condition, we computed the total squared root of Fisher information, and then normalized the corresponding curve in (*A*) by the total information. The normalized curves show high consistency. Under the assumption that the length of the stimulus presentation time modulates the magnitude of the sensory noise (or equivalently, the total resources), without changing the way how coding resources are allocated across orientations, these results suggest that the encoding function is consistently recovered from the data. (*C*) We compare the recovered slope $F^{\prime }\left(\theta \right)$ averaged across conditions (gray solid) with two sources of independent evidence: the reciprocal of discrimination thresholds measured in prior work (53) (dashed), which is expected to be proportional to $F^{\prime }\left(\theta \right)$; and an environmental prior reflecting orientation distributions in images containing natural and human-made content (16) (black solid), which is expected to be proportional to $F^{\prime }\left(\theta \right)$ under efficient coding. All three curves are normalized to have the same average value for comparability. (*D*) The total information $\int \sqrt{J}$ for the five conditions.

We start by estimating the FI from the behavioral responses by fitting the data from individual experimental conditions. Fig. 6*A* plots the recovered FI for each of the five conditions. The recovered FI are higher for the cardinal orientations than the oblique orientations. This result holds for each condition. These results are consistent with the previously reported oblique effect for simple stimuli such as gratings (54), that is, the discrimination threshold for oblique orientations is higher than that of cardinal orientations. To test if the recovered allocation of coding resources is consistent across conditions, we normalized the square root of FI by the total information under each condition. As shown in Fig. 6*B*, the normalized information curves as a function of orientation are generally consistent with each other.

We further test whether the inferred FI from the continuous estimation data is consistent with the efficient coding hypothesis (50), which predicts that the square root of FI should be proportional to the environmental distribution (8, 47, 55). Fig. 6*C* shows that the averaged FI curves that we inferred from the continuous estimation data closely matches the environmental distribution for local visual orientation computed from natural images (from ref. 16), thus providing support for the efficient coding hypothesis. Notably, the inferred FI curve for orientation from estimation data is also consistent with an FI inferred based on threshold measurements obtained from a discrimination task (53).

To study how the information in the orientation code varies with the duration of the stimulus presentation, we quantify the total FI for each condition. We find that overall the integration of visual information over time in this task is sublinear (Fig. 6*D*), i.e., the total FI is substantially lower than that predicted from perfect integration of information over time. Interestingly, the largest increase of information happens between 80 and 160 ms. When comparing the total FI between the 160 and 1,000 ms conditions, the FI for the latter only shows a mild increase. Interestingly, 160 ms is slightly shorter than the typical duration of a fixation for humans in visual search (about 200 to 300 ms). Given the need to plan the next eye movement (56), a plausible interpretation of these results is that the saturation of information beyond 160ms is consistent with the natural time constant for integration of visual information within a single fixation. Note that the Gabor stimuli used in this experiment have a fixed contrast level (18%). In future experiments, it would be interesting to test whether similar results hold for other contrast levels.

#### 1.6.2. Application 2: Identification of the prior and loss function from orientation estimation data.

**Fig. 7.**
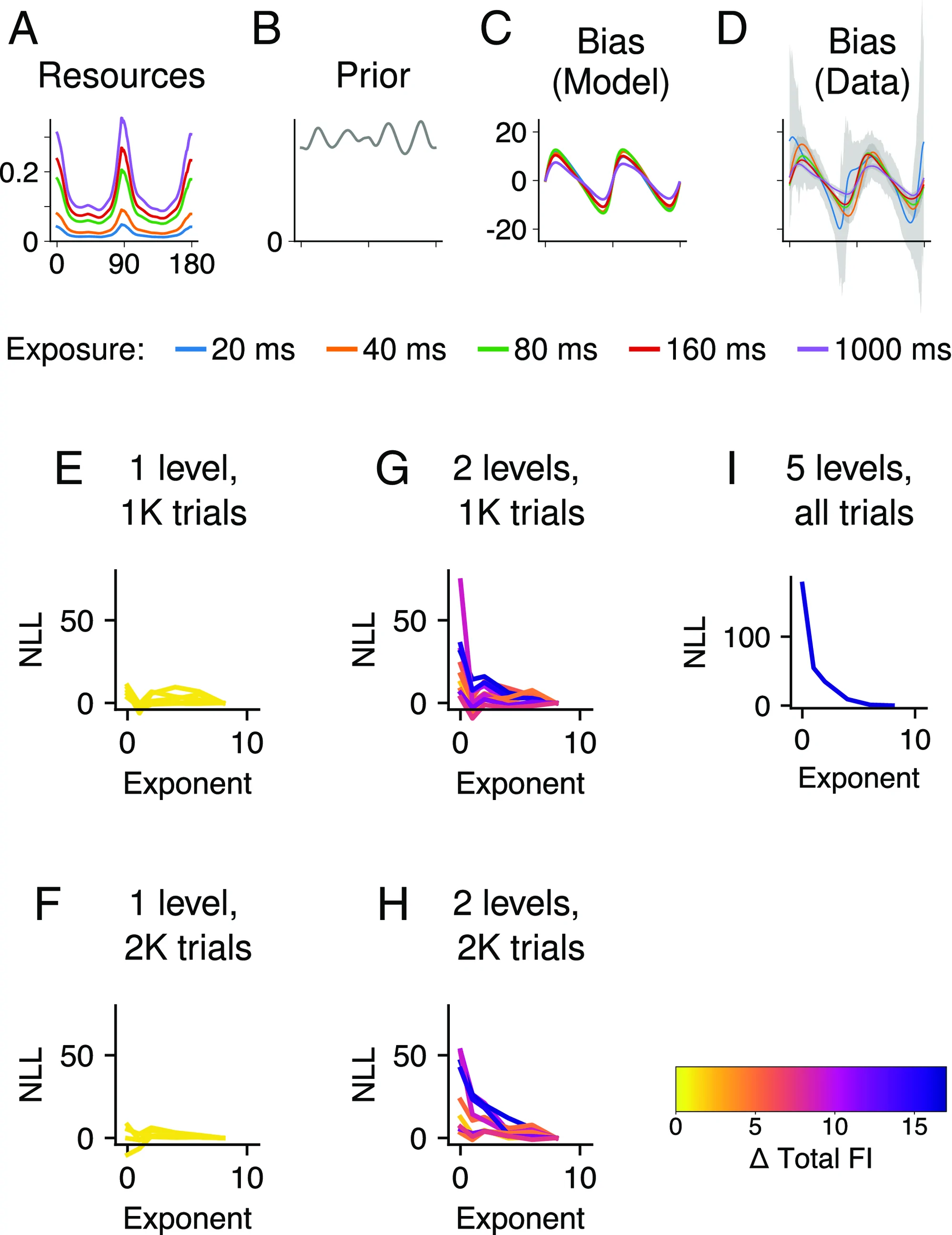
Application to experimental data: recovering the prior and loss function from human estimates of visual orientation. Model identification from human estimation responses for orientation of Gabor arrays [collected by de Gardelle et al. (39)]. (*A* and *B*) Neural resources and the prior inferred from the whole dataset that included data from 5 levels of sensory noise. Fitting recovers periodic resources and a close-to-uniform prior. (*C* and *D*) The best-fit model explains the biases observed in the data. Each color in (*A*, *C*, and *D*) represents a different exposure duration, as indicated in the figure legend. (*E*–*H*) Comparing on subsampled datasets shows that the loss function is hard to identify when only a single noise level is available (yellow color), but becomes easier to identify when two noise levels are combined, at least when these correspond to noise levels with substantially different total FI (dark color). When evaluated on the whole data (9,936 trials), model fit favors higher loss function exponents (e.g., p=8).

We next test the prediction that having behavioral data from multiple noise levels benefits recovery of the Bayesian model. We use the same dataset from the orientation estimation tasks above (39). During these experiments, the experimenters systematically manipulated the lengths of the stimulus presentation time, which should effectively modulate the sensory noise levels (i.e., longer stimulus presentation should reduce noise). We fit the models to data collected from individual conditions and examine the difference in the NLL for models with different loss functions. We find that, even with 2,000 trials, the difference between the best-fitting exponent and worst-fitting exponent of the $L_{p}$ loss function is generally smaller than 5. These results show that it was difficult to identify the loss function from the data collected for a single noise condition (Fig. 7 *E* and *F*) for this task. It follows that the prior distribution is also difficult to identify (*SI Appendix*, Fig. S33).

According to our theory, we expect that the identifiability of the model would increase when combining data from multiple conditions. To test this key theoretical prediction, we fit the model to the combined data from two noise levels together (a total of 10 different combinations). In general, we found that the loss function becomes more identifiable (Fig. 7 *G* and *H*), at least when combining data from noise levels with very different total FI. With 2,000 total trials, the difference between the best-fitting exponent and worst-fitting exponent of the $L_{p}$ loss function can be larger than 20. This difference is much larger than the value obtained from fitting data from a single noise level using the same number of trials. Furthermore, using all data (10k trials) from all five conditions combined, we find the results clearly favor a large exponent in the $L_{p}$ loss function (11), different from the popular squared error loss function considered in prior studies. Finally, the results from combining all data are consistent with the general patterns from combining two noise levels, further supporting that two noise levels might be in principle sufficient for identifiability. Interestingly, the fit to the full data (Fig. 7*A*–*D*) suggests a uniform prior (11), in line with the stimulus distribution within the experiment, whereas the encoding resources are aligned with the long-term environmental statistics, as discussed above. This is consistent with the idea that the prior and encoding may adapt to stimulus statistics at separate timescales (57).

Together, these empirical results are consistent with our theoretical prediction that it is beneficial to collect behavioral data under multiple noise levels experimentally.

#### 1.6.3. Application 3: Difficulty in model identification from time interval data.

We next test the prediction that it would be difficult to identify a class of models with log-normal prior and Weber’s law encoding as mentioned above. This is a special class of models, yet it has been widely used in estimation of magnitudes in previous studies. We will leverage a dataset from Remington et al. (51). In each trial of this experiment, a time interval of a certain duration was presented to the subject, who, upon seeing a visual flash, reproduced the time interval and indicated its end with a key press. The time intervals used were sampled from a bounded range. This task is typical in the study of perception of scalar variables. Similar paradigms were used in other studies (6, 40, 58). Bayesian models have been developed for this task, often under the assumptions that the stimulus encoding satisfied Weber’s law and the prior is Gaussian, log-normal, or uniform in the stimulus range. We previously found that Bayesian model with a log-normal prior outperforms models assuming Gaussian or uniform priors (11). As our theoretical analysis above suggests that this particular class of models may suffer from the issue of nonidentifiability, we seek to test this prediction empirically.

**Fig. 8.**
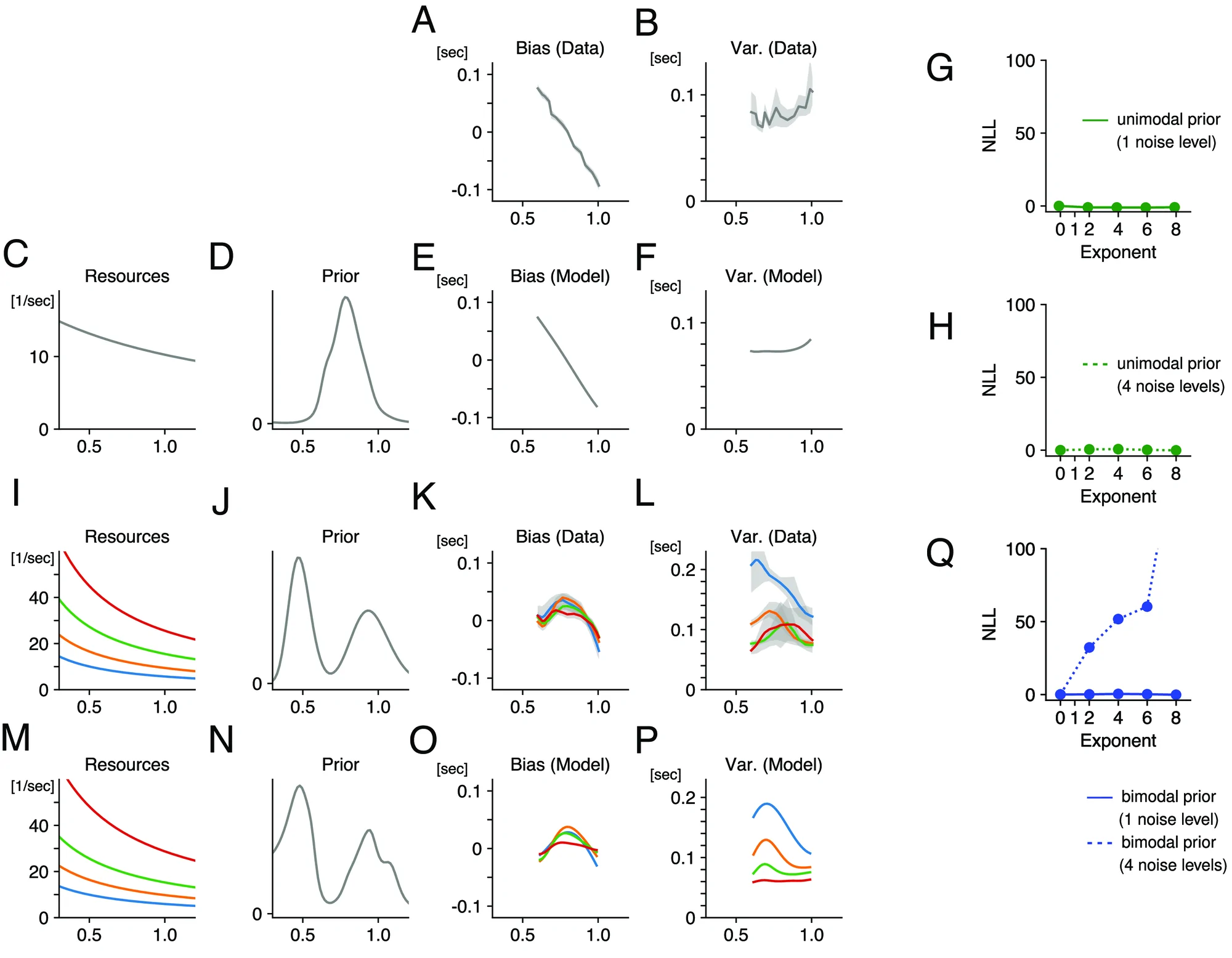
Application to experimental data: estimation of time intervals. (*A*–*H*) Analysis of the data in ref. 51. Remington et al. (51) collected data at one level of sensory noise; all stimuli were from a bounded interval. Behavioral data showing a bias into the interval (*A*), and an increasing variability (*B*). This behavior is well explained in terms of an encoding consistent with Weber’s law (*C*) and a unimodal prior centered in the interval (*D*). We show a fit at p=0, which reproduces the bias and variability (*E* and *F*). However, even when presupposing a Weber’s law-based encoding based on prior work, the loss function cannot be identified, as the same quality of fit is achieved across loss functions (*G*). As the combination of a Weber’s law-based encoding and a lognormal prior is in the exceptional set Ω, the theory suggests that even an experimental design varying the sensory noise might not remedy this. Indeed, when we next simulated data from four noise levels in a model with the same Weber’s law-based encoding and a lognormal prior, the loss function remained unidentifiable (*H*). (*I*–*Q*) Simulation results showing that the nonidentifiability may be resolved using adaptation. We simulated data from four noise levels in a model with the same Weber’s law-based encoding (*I*) but a bimodal prior (*J*), corresponding to inducing a bimodal short-term prior in an adaptation phase preceding the modeled trials. We assume the ground truth loss function is $L_{0}$ loss (p=0). The resulting simulated dataset shows biases and variability (*K* and *L*) clearly different from those produced by the unimodal prior (*A* and *B*). Our theory predicts that the overall model should typically leave the exceptional set Ω when changing the prior, making the model identifiable. Indeed, in this simulated dataset, the loss function can be uniquely identified from the dataset in (*K* and *L*) (dotted blue line in *Q*); at p=0, the prior is recovered (*N*) and the model fits bias and variability (*O* and *P*). In contrast, model fit results for another synthetic dataset (*Q*) with only one level of noise (solid line) cannot single out a loss function, as predicted by our theory.

Fitting the model to the data collected in one condition, we find that the data are well described by a unimodal prior resembling a lognormal prior (Fig. 8*A*–*F*). However, the loss function is difficult to identify. The NLL corresponding to models with different combinations of prior and loss functions are virtually identical based on these data (Fig. 8*G*). These results suggest that while the experimental data are well explained by Bayesian inference based on a log-normal prior operating on a neural representation consistent with Weber’s law, which is consistent with previous results (11), the precise parameter for the log-normal prior and the loss function cannot be identified from these data, consistent with the prediction of our theoretical results.

Remington et al. (51) did not collect the data under multiple noise levels. To further test our theoretical predictions in parameter regimes realistic to the data, we further generated a simulated version of the dataset where the number of noise levels was increased to four (Fig. 8*H*). For this dataset with multiple noise levels, the model remains nonidentifiable. This is expected according to our theory, since the combination of Weber’s law encoding and a lognormal prior is in the exceptional set of unidentifiable models.

If one would like to identify the loss function and the precise prior used by the subjects in this task, how could one resolve this? By our theoretical analysis, a model with the same encoding but a different prior will almost certainly be outside the exceptional set of unidentifiable models. We thus simulated responses from a hypothetical version of the dataset where subjects would have undergone adaptation to a bimodal prior (Fig. 8*I*–*P*) without changing the loss function. Indeed, this simulated dataset makes the loss function well identifiable when multiple noise levels are available (Fig. 8*I*–*Q*), in precise agreement with our theory. These analyses provide a prediction that can be directly tested in future experiments.

## 2. Discussion

Although prior work inferred components of Bayesian observer models from behavioral data, the extent to which these models can be recovered remains poorly understood. A few studies that investigated the identifiability of Bayesian models only focused on a particular set of models under restrictive conditions (17, 19). By systematically investigating the identifiability of a general family of Bayesian models of perception, our results fill an important gap. These results should be applicable to various Bayesian models of continuous one-dimensional stimulus variables, covering a broad class of models considered in visual perception (e.g., refs. 5, 16, and 20), magnitude estimation (e.g., refs. 6, 58, and 59), sensorimotor control (e.g., ref. 3), value-based decision (e.g., refs. 22, 60, and 61), and visual working memory (e.g., refs. 62–64). We acknowledge that there are also other Bayesian models of cognition where our framework is not applicable, such as with discrete or high-dimensional latent variables (e.g., refs. 65–67). Despite their generality, our results can be further extended to even broader settings. Below, we discuss three such directions.

First, our analytical results on identifiability based on measurements at multiple levels require the encoding function to be shared across multiple noise levels. While this seems reasonable, for certain experimental manipulations, e.g., changing the contrast of the stimulus, it is possible that the coding resources (or the discrimination threshold) for the stimulus may scale disproportionally. Thus, it would be useful to understand whether similar results hold when relaxing this assumption. We performed simulations to investigate this question and found that identifiability may be preserved even in models where the encoding varies by noise level (*SI Appendix*, section S5.3).

Second, while our results mainly address the role of sensory noise, experimentally one can also manipulate stimulus noise or both (68). Consider experiments studying the visual orientation perception (16, 49). One can use a stimulus that consists of multiple small Gabor patches with their orientations sampled from a Gaussian distribution. By varying the width of this Gaussian distribution, one can control the magnitude of the stimulus noise (16, 49). Unlike the sensory noise, stimulus noise is external, and cannot be reduced by neural processing. It is of interest to understand whether stimulus noise may benefit model identification. Theoretically, we find that having behavioral responses with nonzero stimulus noise condition in addition to those with zero-stimulus noise (i.e., pure sensory noise) makes most models identifiable, assuming that the encoding is not entirely uniform (*SI Appendix*, section S5.2). Numerically, we also find that varying stimulus noise indeed boosts identifiability. However, when comparing the efficacy of varying the two types of noises (stimulus vs. sensory noises) in improving model identifiability, varying stimulus noise is generally less effective according to simulation results. Further investigation (*SI Appendix*, section S5.2) shows that stimulus noise reduces those components of the biases that are most revealing of the loss function while simultaneously broadening the response distribution, resulting in a less favorable signal-to-noise ratio in identifying the bias from data.

Third, while our results focused on estimation tasks, they may be generalized to another important paradigm in psychophysics, i.e., two-alternative-forced choice tasks (2-AFC). In these tasks, the observers need to compare the test stimulus with a reference to make a binary choice on every trial. A reasonable decision strategy of the observer is to first decode $\hat{\theta }$ and then compare it to the reference (5, 16). We find that, under this decision rule, if both the test stimulus and the reference stimulus are subject to nonnegligible amounts of sensory noise, three levels are sufficient to obtain the same asymptotic guarantee as two levels were under the estimation paradigm (*SI Appendix*, section S5.1.1). If the sensory noise of the reference stimulus is negligible, two noise levels are sufficient, matching the results in estimation paradigm. Interestingly, preliminary results based on numerical simulations suggest that the 2-AFC paradigm might require a larger number of trials than the estimation paradigm in identifying models (*SI Appendix*, section S5.1.2)—an observation that requires further investigation in the future.

In this paper, we have taken the perspective that it is desirable to quantitatively recover all modeling components from the data. Empirically, the desirable notion of identifiability may depend on the question under study. For instance, there may be experiments in which we are primarily interested in understanding how the coding resources are allocated along a particular stimulus dimension, but not the prior distribution or loss function. Also, for cases where the primary goal is to understand the general shape of the prior (i.e., where does the prior peak at), it may be unnecessary to precisely recover it. For these cases, identifiability may be achieved more easily.

While Bayesian models have been popular in modeling perception and cognition, it has been criticized that the results from these models can be difficult to interpret, with unclear theoretical commitments or implications (29, 31). One way to address this criticism is to better understand when the model components of the Bayesian framework can be identified and what kinds of data would be needed to do so. Prior work (17, 19) on the recovery of Bayesian models painted a mixed picture, suggesting that the Bayesian framework has substantial intrinsic degeneracy, though without clear theoretical understanding. Several key differences exist between their work and ours. First, Straub et al. (17) and Acerbi et al. (19) focused on specific functional forms for encoding and prior that accommodate certain types of scalar stimuli. Specifically, Acerbi et al. (19) assume a specific logarithmic form for the encoding and represent the prior as a mixture of Gaussians, whereas we allow both priors and encodings to be arbitrary smooth functions. Acerbi et al. (19) further considered loss functions that are mixtures of two inverted Gaussians, which enabled them to obtain fast numerical solutions for the expected loss, whereas we instead consider the family of $L_{p}$ losses.

Our results, on the one hand, confirm the presence of degeneracy when a single level of sensory noise is considered, as in Acerbi et al. (19). On the other hand, we show that, importantly, this degeneracy can be largely avoided when varying the magnitude of sensory noise. Straub et al. (17) empirically studied identifiability for Bayesian models in the special case of logarithmic encoding and lognormal prior. They numerically found that combining observations from two noise levels alleviated some identifiability limitations, though only loss functions based on $L_{2}$ loss (with an added action cost) were considered. Our findings reveal that this is no accident: for this model, the $L_{p}$ exponent is identifiable only with further strategies such as adaptation to a different prior. In contrast, our findings reveal a substantially broader, and more optimistic, picture. We provide generic identifiability theorems without parametric assumptions on encoding or prior, and without assuming the exponent of $L_{p}$ loss to be known in advance. All three components are usually identifiable separately on the basis of estimation data alone. Notably, our results concern the recovery of model components under the assumption that a dataset is consistent with a Bayesian ideal observer. It is certainly possible that not all behavioral datasets would be consistent with this framework (11). In practice, given a model fitted to behavioral data, one can simulate synthetic data and check if key features, such as biases and variability, are reproduced by the simulated data; if they are not, this would indicate that the dataset is not compatible with a Bayesian ideal observer. Overall, our results represent a step toward increasing the rigor and interpretability of Bayesian models of perception.

It is worth further discussing some key model assumptions for our analytical results. First, our theoretical predictions are derived in the limit of small noise. Numerical simulations suggest that they are valid at realistic noise magnitude, and provide consistent results on data from behavioral experiments. While our theory does not numerically predict which amount of noise is sufficiently small, in practice, one can perform simulations to determine if a given amount of noise is sufficiently small for the identifiability results to apply. Second, our observer model approximates neural noise as Gaussian in sensory space. Under this assumption, the total Fisher information is directly determined by the noise level. As an approximation of neural population code, large neural noise may lead to deviation from effective Gaussian noise (64), making its connection to Fisher information more complicated (69, 70). For large neural noise, the extrinsic representational geometry of the encoding may become important for encoding (64, 71). We discuss the feasibility of extending our theory beyond Gaussian noise to more general sensory noise distributions in *SI Appendix*, section S7.3. Understanding to what extent the representational geometry, and deviations from Gaussian sensory noise, are identifiable from behavior is an interesting problem for future work. Third, regarding the assumption of $L_{p}$ loss functions, in the limit of small noise any smooth symmetric loss function is close to an $L_{p}$ function, though deviations may influence behavior when noise is larger. Simulations suggest that our analysis framework is robust to data generated using the loss functions considered in the simulations of ref. 19 (*SI Appendix*, section S7.4). Asymmetric loss functions (72, 73) might pose further challenges and we leave this to future work.

Our work has direct implications for experimental work that seeks to reverse engineer the perceptual decision process. First, our results on the identifiability of the encoding (i.e., Fisher information) suggest that continuous estimation task is a viable paradigm for studying how the coding resources vary as the stimulus. Most previous studies used 2-AFC to estimate the discrimination threshold (the inverse of the coding resource). Our results suggest that continuous estimation tasks may offer an alternative, sample-efficient strategy for doing so (74). Second, our results provide direct guidance on what would be effective experimental designs if the goal is to infer the components of the Bayesian observers. Our results show that it is useful to have experimental conditions that manipulate the noise characteristics of the stimulus representation. While these manipulations were adopted in some experimental work, their role in model identification is not recognized. Our results show that collecting behavioral responses at multiple noise levels is in fact crucial for identification of Bayesian models, in particular when the loss function is unknown. Further study of this framework may allow the design of experiments that optimize identifiability given a limited number of trials. Third, simulations based on our framework may allow one to investigate whether the model components can be identified practically given a family of models and an experimental design by evaluating the model likelihood obtained based on the simulated data. In addition, the expected precision of the model recovery can also be assessed by comparing the inferred model components with the ground truth used in the simulation. In general, we expect that a better understanding of the identifiability of the Bayesian models (as well as other models used in computational cognitive neuroscience) will facilitate the interpretation of existing data and inform the design of future experiments.

## 3. Materials and Methods

### 3.1. Model of Encoding and Decoding.

We assume a general encoding and decoding model for one-dimensional stimulus variables. Specifically, we assume that the stimulus space X is a circle or a bounded interval, the space of measurement (the neural encoding signal in the brain) is denoted as Y, and the map F:X→Y is a bijection. Formally, the neural encoding can be described by the following equation

[4] $$\begin{array}{c} m=F(\theta )+\epsilon . \end{array}$$

We assume, without loss of generality, that Y has volume 1. Given a particular loss function ℓ and an encoding model m∈Y, the Bayesian estimator $\hat{\theta }\in X$ that minimizes the loss function is obtained as

[5] $$\begin{array}{c} \hat{\theta }:=\;\arg_{\hat{\theta }\in X}\min\int_{X}\ell \left(\hat{\theta },\theta \right)p\left(\theta |m\right)d\theta . \end{array}$$

At a stimulus θ, the estimator $\hat{\theta }$ has bias $E[\hat{\theta }]-\theta$.

The resource allocation is $\sqrt{J(\theta )}=\frac{F^{\prime }\left(\theta \right)}{\sigma }$, which is proportional to the slope $F^{\prime }\left(\theta \right)$ of the encoding function, with proportionality factor given by the reciprocal of the sensory noise SD. We study model variants where noise may also enter *before* the map F(·) in *SI Appendix*, section S5.2.

### 3.2. Formalizing the Space of Models.

Let F(X) be the set of real-valued functions on X that are five times continuously differentiable, whose values are always strictly positive, and where ∫f=1. Both the prior $p_{\mathrm{prior}}$ and the resource allocation $F^{\prime }\left(\theta \right)$ are elements of F(X).

We take P⊂N to be a finite set of exponents p for $L_{p}$ loss functions; we assume P⊂{0,1,2,4,6,⋯} for our theoretical results.

The space of encoding models is then the product space M:=F×F×P, whose elements are tuples of priors $p_{\mathrm{prior}}$, resource allocations $F^{\prime }$, and loss function exponents p.

We quantify volume on the infinite-dimensional space M by parameterizing both $p_{\mathrm{prior}}$ and $F^{\prime }$ in terms of their logarithms, and placing a Gaussian process measure on these. Independently sampling prior, encoding, and loss function determines a notion of measure or volume on the space of models. See *SI Appendix*, section S2.1 for details. The key here is that prior and encoding are sampled independently, so that there are no systematic algebraic relations between prior and encoding across the stimulus space. Our statement below that the exceptional set Ω has volume zero intuitively means that a sampled model is extremely unlikely to lie in Ω.

### Identifiability Theorems.

We consider the response distribution P(y|M,θ,σ) determined by the model M∈M, θ∈X, and a sensory noise magnitude σ>0. We assume that these distributions are known at each stimulus θ, at one or more sensory noise levels. The noise magnitudes σ associated with the noise levels are assumed to be unknown, and inferred together with the other model components.

Our first theorem concerns recovery of the encoding, providing an explicit formula for the Fisher information in terms of the overlap in response distributions:

Theorem 3.1 *(Recovering Encoding from Response Distribution) In the absence of motor noise, the encoding* F *and the sensory noise magnitude can be recovered from the response distribution at a single level of sensory noise, with estimation error exponentially small in* $\frac{1}{\sigma }$:

$$\begin{array}{c} \sqrt{J(\theta )}=\sqrt{4\pi }\cdot \frac{\text{d}}{\text{d}h}P\left(\hat{\theta }\left(\theta +h\right)>\hat{\theta }\left(\theta \right)\right)+O\left(\exp\left(-1/\sigma \right)\right), \end{array}$$

*where the derivative is evaluated at* h=0, *and* $\hat{\theta }\left(\theta +h\right)$ *and* $\hat{\theta }\left(\theta \right)$ *are independent response samples at stimuli* θ+h *and* θ, *respectively*.

The proof of this theorem is in *SI Appendix*, section S2.4. Numerical approximation of this formula allows straightforward estimation, independent of the loss function, as we illustrate in *SI Appendix*, Fig. S3.

Our next theorem concerns recovery of the prior conditional on the loss function. It guarantees recovery of the prior when the loss function is known:

Theorem 3.2 *(One Level of Sensory Noise) Let* σ>0 *be the SD of sensory noise, and* p∈P. *Assume the response distribution* $P(\hat{\theta }|M,\theta ,\sigma )$ *is given at each* θ, *under a ground-truth model* $\langle F^{\prime },p_{\mathrm{prior}},p\rangle \in M$. *There is a functional* $\Phi_{p}$ *mapping these distributions to* $\langle {\hat{F}}^{\prime },\hat{p_{\mathrm{prior}}},p\rangle \in M$ *such that*

$$\begin{array}{cc} |\hat{F}\left(\theta \right)-F\left(\theta \right)| & =O(\sigma^{2}),\\ |\hat{p_{\mathrm{prior}}}\left(\theta \right)-p_{\mathrm{prior}}\left(\theta \right)| & =O(\sigma^{2}), \end{array}$$

*as* σ→0. *Constants in* O(·) *depend on* F *and* $p_{\mathrm{prior}}$, *but not* θ.

We next state our identifiability theorem for the more general setup where encoding, prior, and loss function are all unknown a priori. We use Ω to denote the exceptional set of unidentifiable models.

Theorem 3.3 *(Two Levels of Sensory Noise) There is a subset* Ω⊂M *of volume* 0 *such that the following holds. Let* $0<\sigma_{1}<\sigma_{2}$ *be two levels of sensory noise. Assume the response distributions* $P(\hat{\theta }|M,\theta ,\sigma_{i})$ *are given at each* θ *and for both* $\sigma_{1},\sigma_{2}$, *under a ground-truth model* $M=\langle F^{\prime },p_{\mathrm{prior}},p\rangle \in M$. *There is a functional* Φ *mapping the collection of these distributions to* $\langle {\hat{F}}^{\prime },\hat{p_{\mathrm{prior}}},q\rangle \in M$ *such that—provided* M∉Ω*—we have*

$$\begin{array}{cc} |\hat{F}\left(\theta \right)-F\left(\theta \right)| & =O(\sigma_{2}^{2}),\\ |\hat{p_{\mathrm{prior}}}\left(\theta \right)-p_{\mathrm{prior}}\left(\theta \right)| & =O(\sigma_{2}^{2}),\\ q & =p\;\text{for}\;\sigma_{2}^{2}\;\text{small}. \end{array}$$

*Constants in the* O(·) *expressions depend on the ratio* $\frac{\sigma_{2}}{\sigma_{1}}$, *and on* F *and* $p_{\mathrm{prior}}$, *but not on* θ.

This theorem provides in-principle identification of the loss function in all models outside of Ω. Importantly, this theorem does not presuppose that the magnitudes $\sigma_{1},\sigma_{2}$ are known. It only requires that $\sigma_{1},\sigma_{2}$ are small and distinct. Although in theory, two levels of noise is sufficient, practically, it may be advantageous to obtain observations at a larger number of noise levels across different scales according to our numerical results.

Theorem 3.4 *(Role of Motor Noise) Theorem 3.3 is not affected by adding symmetric isotropic motor noise of variance* $\tau^{2}$, *and by guessed responses appearing at a rate* 0<γ<1. *The constants in the* O(·) *expressions then depend additionally on the ratio* $\frac{\tau }{\sigma_{1}}$ *and on* γ.

### Implementation.

We use the fitting procedure introduced in ref. 11, which, given a loss function, jointly fits encoding, prior, motor and sensory noise magnitudes, and guessing rate, by maximizing the data’s likelihood under the model. Prior and encoding are specified on a discretized grid over X. Following ref. 11, its size is 180 in the models in figures 1–7, and 200 in the model in Fig. 8. Following ref. 11, we implement the $L_{p}$ loss function on circular spaces via powers of the cosine ($\ell \left(x,y\right):={\left(1-\cos\left(x-y\right)\right)}^{p/2}$), as they found this to provide better fit to behavioral data than a version based on absolute distance. In the low-noise setting, $\ell \left(x,y\right)∼{\left|x-y\right|}^{p}$, i.e., our theoretical results apply to this implementation.

We provide a description of the practical model fitting procedure in *SI Appendix*, section S8. See *SI Appendix* for a full version of Materials and Methods, including information on Simulations and Experimental Data.

### AI Usage.

We used GPT-5.4 for supporting the preparation of plotting and LaTeX formatting code, and for brain-storming, proof-reading, and refining mathematical material in *SI Appendix*. The authors reviewed all AI-assisted output and take full responsibility for the content of the paper.

## Supplementary Material

Appendix 01 (PDF)

## Data Availability

No new experimental datasets were collected in this study. The experimental datasets used in this study were all previously published (39, 51) and are publicly available. Simulated datasets are available, together with the source code, at https://github.com/m-hahn/identifiability-bayesian-models.
